# Ki-67 Quantification in Breast Cancer: A Comparison of Hotspot and Global Scoring Methods Using a Standardized Android Application

**DOI:** 10.7759/cureus.107583

**Published:** 2026-04-23

**Authors:** Meet Patel, Sneha Jawalkar

**Affiliations:** 1 Pathology, Shri B. M. Patil Medical College, Hospital and Research Centre, BLDE (Deemed to be University), Vijayapura, IND

**Keywords:** breast carcinoma, hotspot method, ikwg, ki-67 proliferation index, weighted-global method

## Abstract

Background

Ki-67 is an important proliferative biomarker in breast carcinoma; however, methodological variability in its assessment limits reproducibility and clinical applicability. The International Ki-67 in Breast Cancer Working Group (IKWG) has proposed a standardized global scoring method with a freely available Android application to address this issue. This study aimed to compare hotspot and weighted-global Ki-67 scoring methods using the IKWG Android application in invasive breast carcinoma, assess their correlation and categorization, and evaluate their diagnostic performance in predicting histological grade, with potential relevance to clinical decision-making and treatment stratification.

Methodology

A hospital-based, cross-sectional study was conducted on 53 cases of invasive breast carcinoma. Ki-67 immunohistochemical slides were digitally scanned and scored using both hotspot and weighted-global methods via the IKWG Android application. Statistical analysis was performed using the Mann-Whitney U test, Spearman’s correlation, and receiver operating characteristic analysis.

Results

The hotspot method produced a markedly elevated mean Ki-67 index (57.1 ± 22.6%) in contrast to the weighted-global method (43.1 ± 24.0%; p = 0.004). Using IKWG cutoffs, 44 (83.0%) tumors were classified as high proliferative by hotspot versus 36 (67.9%) by weighted-global scoring. Both methods showed a very strong correlation (Spearman’s r = 0.954, p < 0.001). The weighted-global Ki-67 demonstrated excellent diagnostic performance for predicting high-grade tumors (area under the curve = 0.991), with 100% specificity and 84.9% overall accuracy. Grade 2 tumors predominated (26 (49.1%)), followed by Grade 3 (18 (34.0%)) and Grade 1 (9 (17.0%)).

Conclusions

Hotspot scoring systematically overestimates tumor proliferation. The weighted-global method, incorporating intratumoral heterogeneity, provides a more balanced and biologically representative Ki-67 estimate. Standardization via the IKWG Android application improves reproducibility and supports reliable clinical decision-making.

## Introduction

Breast cancer remains the most frequently diagnosed malignancy and the leading cause of cancer-related mortality among women globally, with approximately 2.3 million new cases reported annually [1]. Its heterogeneous biology necessitates comprehensive biomarkers to guide therapeutic stratification. Among these, Ki-67, a nuclear protein expressed in all active phases of the cell cycle (G1, S, G2, and mitosis) but absent in quiescent G0 cells, has become the most widely utilized proliferation marker in breast cancer management [2,3]. First identified in the early 1980s, Ki-67 is consistently associated with adverse prognostic features, including larger tumor size, higher histological grade, lymph node positivity, and hormone receptor negativity [4,5].

Despite its clinical relevance, Ki-67 assessment is hampered by significant inter-observer and inter-laboratory variability arising from pre-analytical, analytical, and scoring methodology-related factors [6,7]. Standardized global scoring provides more consistent prognostic value than localized hotspot methods [8].

The International Ki-67 in Breast Cancer Working Group (IKWG) was established to develop these standardized approaches. The IKWG global method involves estimating the proportion of tumors showing negligible, low, medium, or high Ki-67 staining intensities to generate a weighted score [7,9]. This approach addresses the limitations of the conventional hotspot method, which focuses solely on areas of peak proliferative activity and may overestimate tumor aggressiveness [10]. The present study directly compared both methods using the IKWG Android application to evaluate standardized Ki-67 assessment in routine pathology practice [9,11].

## Materials and methods

Study design and patient selection

This cross-sectional study was conducted in the Department of Pathology at BLDE (Deemed to be University), Shri B. M. Patil Medical College, Hospital, and Research Centre, Vijayapura, India, on 53 cases of invasive breast carcinoma that underwent modified radical mastectomy. The sample size was calculated using a proportion-based formula; however, the study was not specifically powered for comparative analysis between the two methods. The study was conducted between March 2024 and October 2025 after obtaining approval from the Institutional Ethics Committee (approval number: BLDE(DU)/IEC-SBMPMC/129/2023-24). Exclusion criteria included the following: (1) cases with prior neoadjuvant chemotherapy or hormonal therapy, and (2) tumors other than invasive breast carcinoma. Relevant clinicopathological data were obtained from pathology records and medical case files.

Tissue processing, histopathological evaluation, immunohistochemistry, and digital scanning

All mastectomy specimens were fixed in 10% neutral buffered formalin and processed using standard histopathological techniques. Sections of 4-5 µm in thickness were cut from the representative paraffin-embedded blocks. One section was stained with hematoxylin and eosin for morphological assessment and histological grading according to the modified Scarff-Bloom-Richardson system. The second section was mounted on poly-L-lysine-coated slides for Ki-67 immunohistochemistry using the MIB-1 clone. Standard heat-induced antigen retrieval was performed by a horseradish peroxidase-polymer detection system and visualization with 3,3'-diaminobenzidine [12]. The slides were digitally scanned using Motic Easy Scan software, and the images were reviewed on the Aperio ImageScope platform.

Ki-67 quantification protocol

In the present study, Ki-67 scoring was performed by a single observer to maintain consistency in evaluation and minimize inter-observer variability. While formal calibration exercises and blinding were not specifically undertaken, standard assessment protocols were followed throughout the analysis. Ki-67 immunostaining was quantified using both hotspot and weighted-global methods with the IKWG Android visual scoring application on digital whole-slide images [9,11].

**Figure 1:**
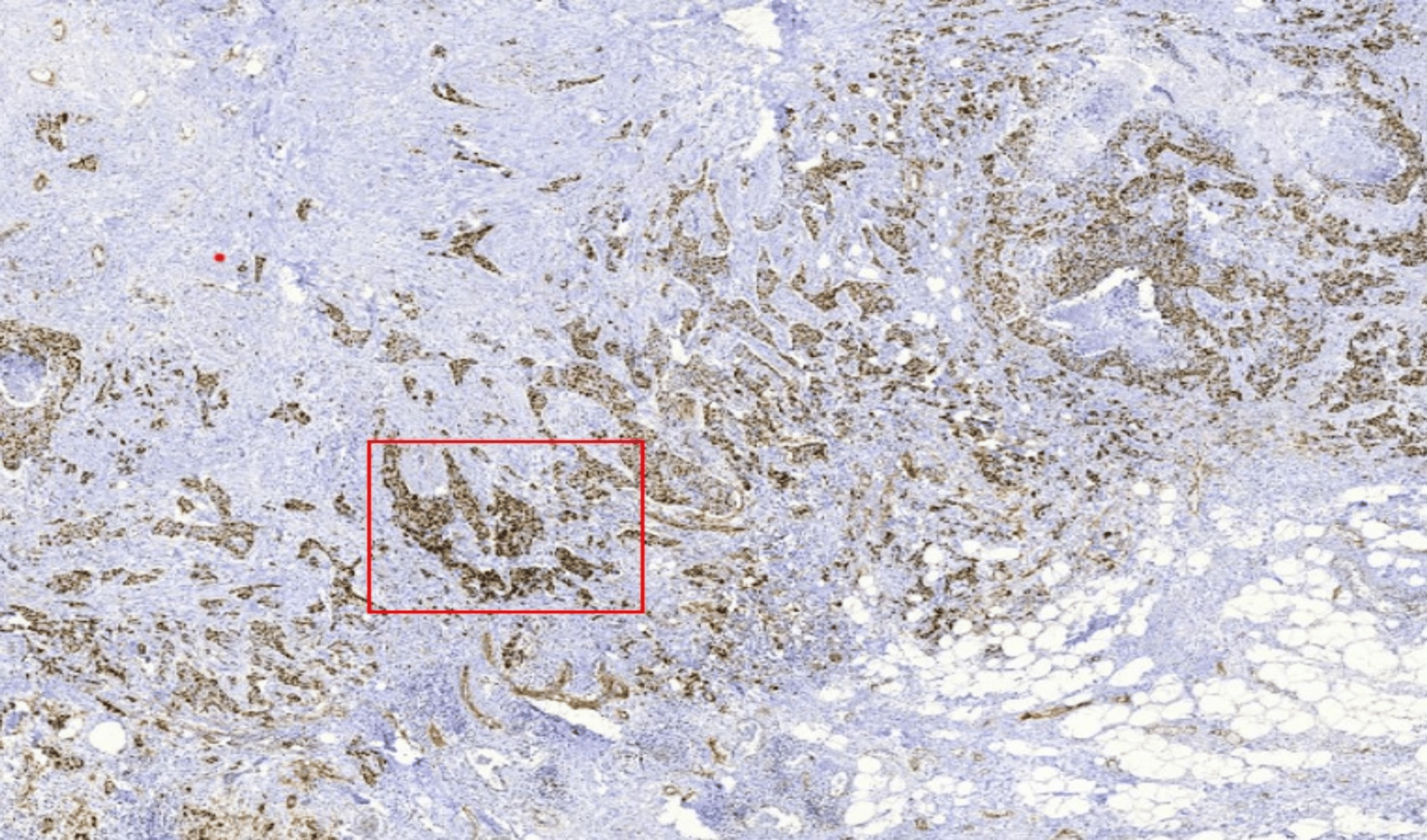
Immunohistochemical Ki-67 scanner view with the hotspot area (box).

**Figure 2:**
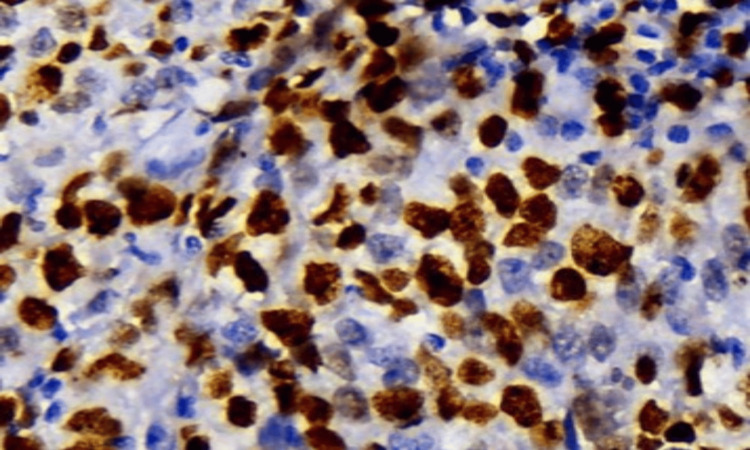
Digital immunohistochemical image of Ki-67 (400×) hotspot area with 500 nuclei counted.

**Figure 3:**
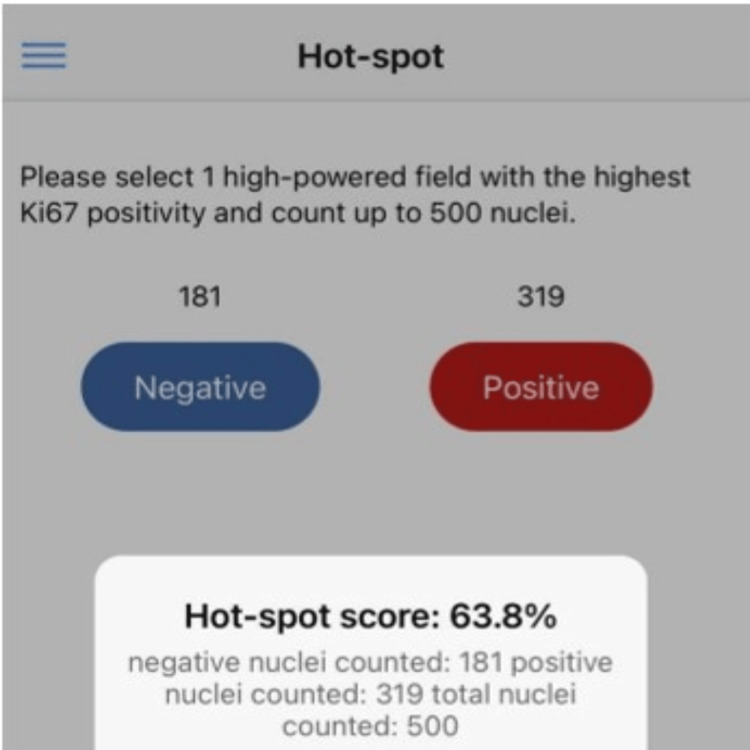
Ki-67 Android scoring app showing 500 nuclei are entered manually.

For the hotspot method, the entire tumor section was initially scanned at low magnification (10×) to identify the area showing the highest density of Ki-67-positive tumor cells. A minimum of 500 tumor cell nuclei were then manually counted within this hotspot region, and the counts were entered into the application to obtain the Ki-67 index (Figures 1-3). To minimize counting fatigue and reduce the risk of recounting or selection bias, hotspot areas were carefully delineated and marked before manual counting.

**Figure 4:**
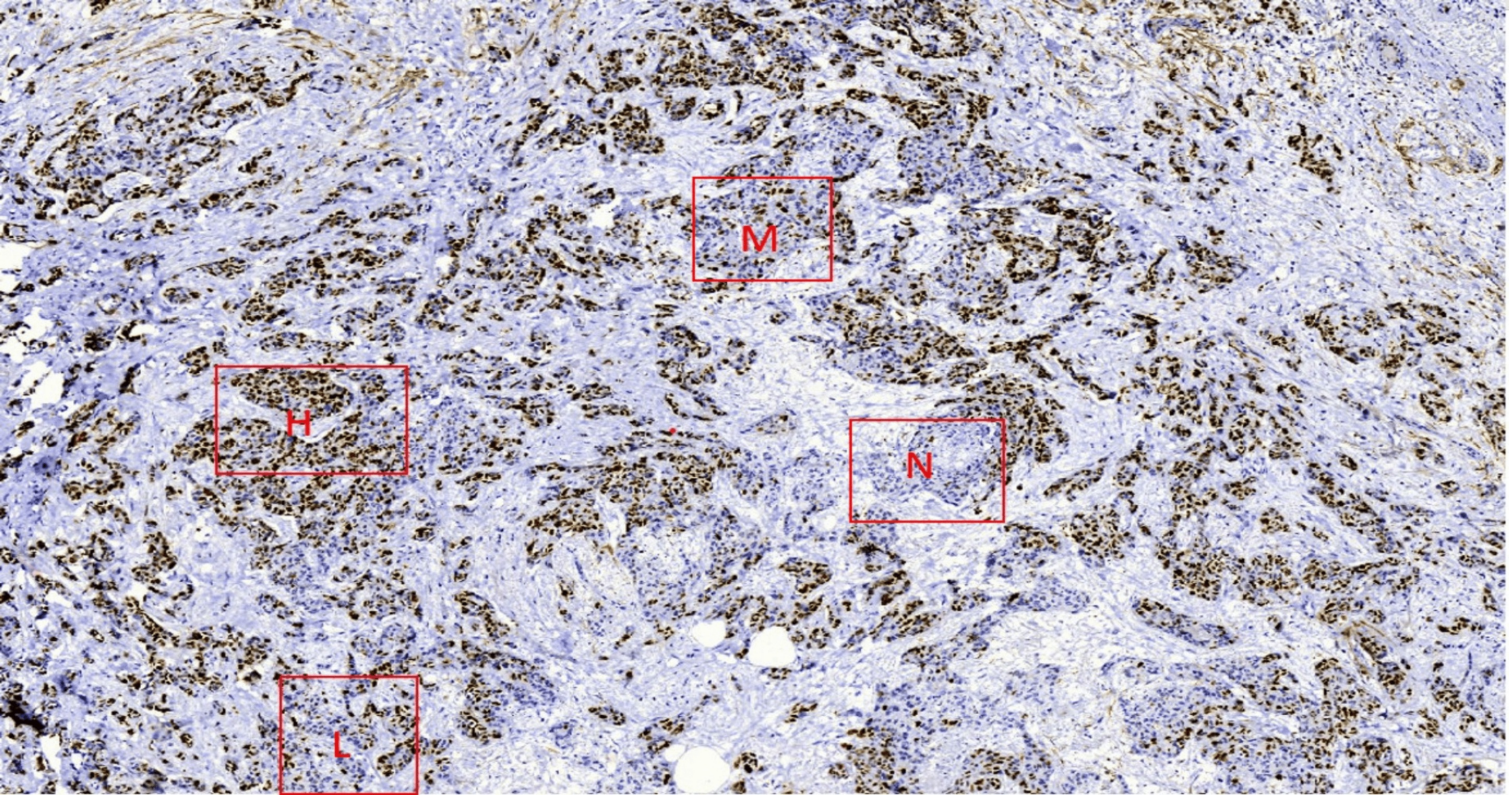
Digital image of whole-slide scanner view with boxes highlighting areas with different Ki-67 scores (negligible, low, medium, high).

For the weighted-global method, the entire tumor section was first evaluated at low magnification to estimate the proportional distribution of areas showing negligible, low, medium, and high Ki-67 staining. Up to four representative fields corresponding to these staining categories were selected in proportion to their area contribution (Figure 4).

**Figure 5:**
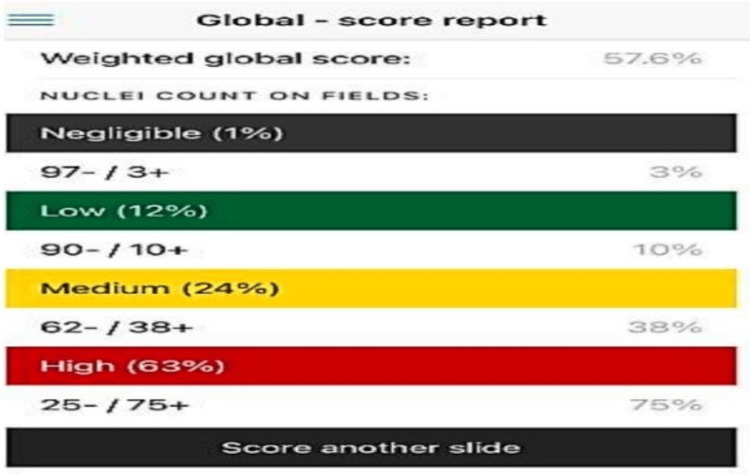
Ki-67 Android app: global-weighted score report.

In each selected field, 100 tumor cell nuclei were counted at high magnification (400×) using a systematic typewriting pattern, and the counts were entered into the application to obtain the Ki-67 index. The application subsequently generated both an unweighted global score and a weighted global score by incorporating the proportional contribution of each staining category [13,14] (Figure 5).

Statistical analysis

All data were entered into Microsoft Excel (Microsoft Corp., Redmond, WA, USA), and statistical analysis was performed using IBM SPSS Statistics for Windows, version 29.0 (IBM Corp., Armonk, NY, USA). Continuous variables were expressed as mean ± standard deviation and median with percentiles. The Mann-Whitney U test was used to compare Ki-67 indices obtained by the two methods. Spearman’s rank correlation coefficient was applied to assess the correlation between hotspot and weighted-global Ki-67 scores. Receiver operating characteristic (ROC) curve analysis was performed to evaluate the diagnostic performance of the weighted-global method in predicting histological grade. A p-value <0.05 was considered statistically significant.

## Results

Patient demographics and clinicopathological profile

The study included 53 female patients diagnosed with invasive breast carcinoma. The highest proportion of cases occurred in the 61-70-year age group (23 (43.4%)), followed by the 51-60-year group (13 (24.5%)). Regarding tumor characteristics, the majority of tumors were classified as pT2 (32 (60.4%)). Lymph node metastasis was identified in 39 (73.6%) cases, corresponding to nodal stages pN1-pN3, while 14 (26.4%) patients had no nodal involvement (pN0). Histologically, Grade 2 tumors constituted the largest proportion of cases (26 (49.1%)), followed by Grade 3 (18 (34.0%)) and Grade 1 tumors (9 (17.0%)) (Table 1).

**Table 1:** Clinicopathological profile of the study population (n = 53).

Variable	Category	n (%)
Age group (years)	<40	7 (13.2%)
	41–50	6 (11.3%)
	51–60	13 (24.5%)
	61–70	23 (43.4%)
	>70	4 (7.5%)
Histological grade	Grade 1	9 (17.0%)
	Grade 2	26 (49.1%)
	Grade 3	18 (34.0%)
pT category	pT1	3 (5.7%)
	pT2	32 (60.4%)
	pT3	10 (18.9%)
	pT4	8 (15.1%)
pN category	pN0	14 (26.4%)
	pN1	17 (32.1%)
	pN2	10 (18.9%)
	pN3	12 (22.6%)

Statistical comparison of Ki-67 scoring

The mean Ki-67 index by the hotspot method was 57.1%, with a median of 59% and a standard deviation of 22.6%. In comparison, the weighted-global method showed a lower mean value of 43.1%, with a median of 42.4% and a standard deviation of 24.0%.

Ki-67 categorization using hotspot and weighted-global methods

Applying the IKWG classification, the hotspot method categorized 44 (83.0%) cases as high proliferative (≥30%), 8 (15.1%) as intermediate (6-29%), and 1 (1.9%) as low proliferative (≤5%). In comparison, the weighted-global method classified 36 (67.9%) tumors as high proliferative, 14 (26.4%) as intermediate, and 3 (5.7%) as low proliferative (Table 2).

**Table 2:** Ki-67 category distribution.

Ki-67 category	Hotspot method (n = 53), n (%)	Weighted-global method (n = 53), n (%)
Low (≤5%)	1 (1.9%)	3 (5.7%)
Intermediate (6–29%)	8 (15.1%)	14 (26.4%)
High (≥30%)	44 (83.0%)	36 (67.9%)

Receiver operating characteristic curve analysis and diagnostic performance

ROC curve analysis was performed to evaluate the ability of the weighted-global Ki-67 score to predict high-grade tumors. For the purpose of ROC analysis and evaluation of diagnostic performance, histological grade was treated as a binary variable, with Grade 3 tumors considered as high-grade and Grade 1 and Grade 2 tumors grouped together as low/intermediate grade.

The analysis demonstrated excellent diagnostic performance with an area under the curve (AUC) of 0.991 (95% confidence interval (CI) = 0.973-1.000; p < 0.001). The weighted-global method showed a sensitivity of 52.9%, specificity of 100% (95% CI = 90.26-100.00%), and an overall diagnostic accuracy of 84.9% (n = 45/53; 95% CI = 72.41-93.2%) (Table 3).

**Table 3:** Receiver operating characteristic (ROC) curve analysis.

ROC performance parameters	Value	95% confidence interval
Area under the curve	0.991	0.973–1.000
Sensitivity (%)	52.94	27.81–77.02
Specificity (%)	100.00	90.26–100.00
Accuracy (%)	84.91	72.41–93.25

## Discussion

The present study demonstrated a statistically significant difference between hotspot and weighted-global Ki-67 indices (mean = 57.1% vs. 43.1%; p = 0.004), consistent with previous reports [15,16]. The hotspot method evaluates the most proliferatively active tumor areas and therefore yields higher Ki-67 values, particularly in tumors with marked intratumoral heterogeneity. Similar observations were reported by Angel et al. [15], who found higher Ki-67 values with hotspot assessment (42.4%) compared with global scoring (37.13%).

In contrast, the weighted-global method incorporates the proportional contribution of different tumor regions, providing a more representative estimate of overall proliferative activity. In the present study, the median Ki-67 value obtained by the hotspot method (59.0%) was substantially higher than that of the weighted-global method (42.4%). Comparable findings were reported by Jang et al. [16], who observed higher median Ki-67 values with hotspot scoring compared with the average method (18.5% vs. 13.0%).

The distribution of tumors across Ki-67 proliferative categories varied depending on the scoring method used. Using the hotspot technique, 44 (83.0%) cases were categorized as high proliferative (≥30%), whereas the weighted-global method classified 36 (67.9%) tumors in this category. Hotspot scoring classifies a greater proportion of tumors as high proliferative. In contrast, the weighted-global method provides a more balanced distribution across proliferative categories. Angel et al. reported similar findings, demonstrating that hotspot scoring tends to produce higher Ki-67 categorization using St. Gallen cut-offs [15].

The IKWG recommends that Ki-67 values ≥30% indicate high proliferative activity, whereas values between 6% and 29% represent an equivocal range that should not be interpreted in isolation [11]. By shifting more tumors into the intermediate category, the weighted-global method aligns more closely with IKWG recommendations and reduces the risk of treatment escalation due to hotspot-inflated Ki-67 values.

In the present study, the weighted-global Ki-67 method demonstrated excellent diagnostic performance, with an AUC of 0.991, specificity of 100%, and a sensitivity of 52.9%. The high specificity indicates that the weighted-global method reliably identified low-grade tumors within the study cohort. However, the relatively lower sensitivity suggests that a proportion of high-grade tumors were not identified by this method. This reflects a potential risk of underestimation of high-grade tumors when relying solely on the weighted-global Ki-67 approach. Therefore, while the weighted-global method shows strong diagnostic performance, its moderate sensitivity indicates that results should be interpreted in conjunction with histopathological grading and other relevant parameters.

Despite numerical differences, both methods showed a very strong correlation (r = 0.954, p < 0.001), indicating that both approaches ranked tumors similarly in terms of proliferative activity. Angel et al. [15] reported excellent interobserver reproducibility for the weighted-global Ki-67 method (intraclass coefficient (ICC) = 0.967) [15]. Similarly, Radhakrishnan et al. [10] demonstrated higher reproducibility with the global method (ICC = 0.971) compared with hotspot scoring (ICC = 0.819), supporting the greater consistency of the global approach (Table 4).

**Table 4:** Comparative analysis of Ki-67 assessment metrics across studies. IKWG = International Ki-67 in Breast Cancer Working Group; ROC = receiver operating characteristic; AUC = area under the curve; ICC = intraclass coefficient; NR = not reported

Comparison parameter	Present study (n = 53)	Angel et al. [15]	Jang et al. [16]	Radhakrishnan et al. [10]
Ki-67 index (%)				
Hotspot (mean/median)	57.10/59.0	42.40/NR	NR/18.5	NR
Global (mean/median)	43.14/42.4	37.13/NR	NR/13.0	NR
High proliferative group (%)				
Hotspot method	83.0% (IKWG guidelines)	70.0% (St. Gallen guidelines)	NR	NR
Global method	67.9% (IKWG guidelines)	63.3% (St. Gallen guidelines)	NR	NR
Diagnostic performance (ROC)				
AUC	0.991	NR	0.700–0.711	NR
Specificity	100%	NR	NR	NR
Statistical reliability				
Inter-method correlation (r)	0.954	NR	NR	NR
Reproducibility (ICC)	NR	0.967	NR	0.819–0.971
Efficiency and stage				
Assessment time (hotspot)	3–5 minutes	4–5 minutes	NR	NR
Assessment time (global)	5–7 minutes	5–6 minutes	NR	NR

The hotspot method required three to five minutes per case, compared with five to seven minutes for the weighted-global method; this modest additional time is justified by improved reliability.

The findings of this study have direct clinical implications, as overestimation of Ki-67 by hotspot methods may lead to unnecessary escalation of therapy. Adoption of standardized weighted-global scoring can improve treatment stratification and reduce overtreatment.

Limitations

The present study has some limitations. The sample size was relatively small (n = 53), and the study was conducted at a single center, which may limit generalizability and preclude assessment of inter-laboratory variability. In addition, the lack of long-term follow-up prevented evaluation of prognostic significance. An important methodological limitation is the absence of a formal inter-observer reproducibility assessment, as Ki-67 scoring was performed by a single observer. In addition, the selection of hotspot regions and fields for weighted-global assessment may introduce observer-dependent variation, potentially influencing the results, despite the use of a standardized IKWG-based application.

## Conclusions

The findings of the present study demonstrate differences between hotspot and weighted-global Ki-67 scoring methods using a standardized approach. While these methods may contribute to more consistent assessment, the study design does not allow definitive conclusions regarding reproducibility or clinical decision-making. These observations support the potential incorporation of standardized Ki-67 scoring into reporting frameworks to enhance uniformity. However, further multi-institutional studies incorporating inter-observer analysis and clinical outcome correlation are required to validate these findings, refine clinically relevant cut-off values, and strengthen the prognostic and predictive utility of Ki-67.
